# ECG-based machine-learning algorithms for heartbeat classification

**DOI:** 10.1038/s41598-021-97118-5

**Published:** 2021-09-21

**Authors:** Saira Aziz, Sajid Ahmed, Mohamed-Slim Alouini

**Affiliations:** King Abdullah University of Science and Technology, Thuwal, Saudi Arabia

**Keywords:** Cardiology, Diseases, Health care, Electrical and electronic engineering

## Abstract

Electrocardiogram (ECG) signals represent the electrical activity of the human hearts and consist of several waveforms (P, QRS, and T). The duration and shape of each waveform and the distances between different peaks are used to diagnose heart diseases. In this work, to better analyze ECG signals, a new algorithm that exploits two-event related moving-averages (TERMA) and fractional-Fourier-transform (FrFT) algorithms is proposed. The TERMA algorithm specifies certain areas of interest to locate desired peak, while the FrFT rotates ECG signals in the time-frequency plane to manifest the locations of various peaks. The proposed algorithm’s performance outperforms state-of-the-art algorithms. Moreover, to automatically classify heart disease, estimated peaks, durations between different peaks, and other ECG signal features were used to train a machine-learning model. Most of the available studies uses the MIT-BIH database (only 48 patients). However, in this work, the recently reported Shaoxing People’s Hospital (SPH) database, which consists of more than 10,000 patients, was used to train the proposed machine-learning model, which is more realistic for classification. The cross-database training and testing with promising results is the uniqueness of our proposed machine-learning model.

## Introduction

**Figure 1:**
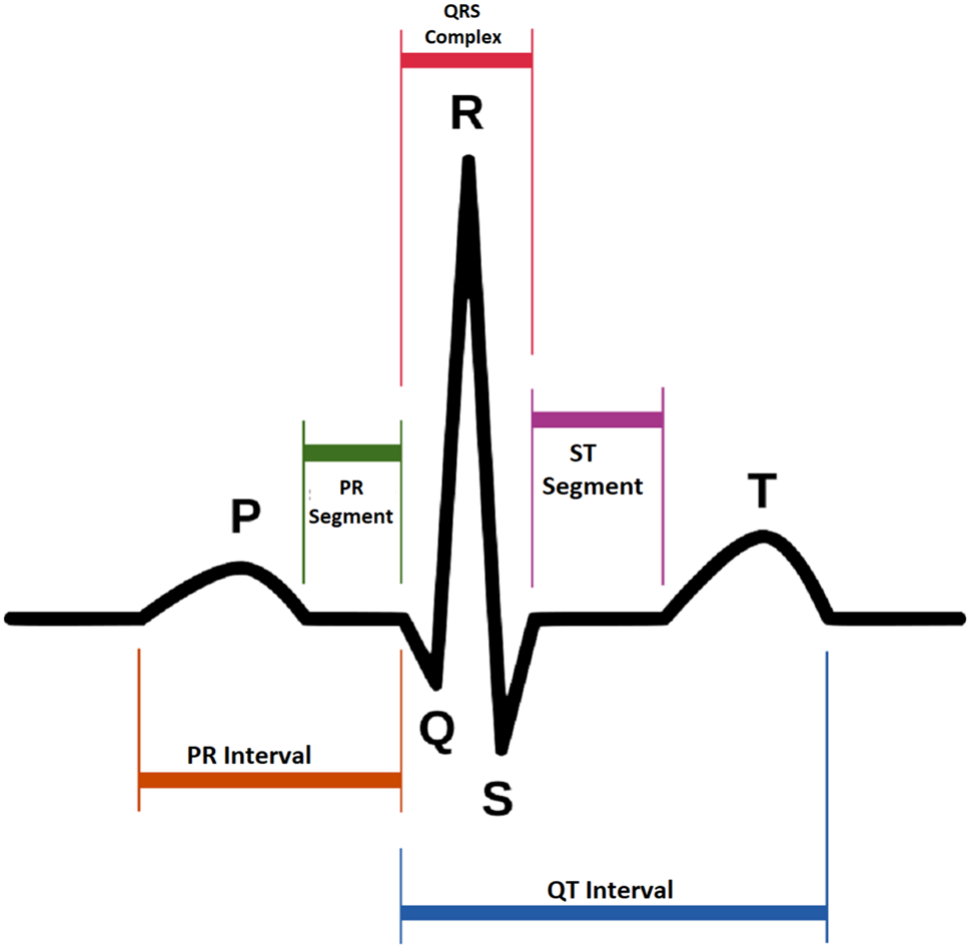
Morphology of a normal ECG.

According to the World Health Organization, cardiovascular diseases (CVDs) are the leading cause of death globally^1^. In recent years, various programs and policies have been implemented in increasingly diverse communities to provide tools, strategies, and other best practices for reducing the incidences of initial and recurrent cardiovascular events. To achieve this goal, the electrocardiogram (ECG) has become the most commonly used biosignal for the prompt detection of CVDs. The ECG is a graphical representation of heart electrical activity, and it is used to identify various heart diseases and abnormalities^2^. Doctors have been using ECG signals to detect heart diseases such as arrhythmia and myocardial infarctions for over 70 years. An ECG signal consists of P, QRS complex, and T waves^3–5^, as shown in Fig. 1. Additionally, a U wave may be present. By analyzing the variations of these waves, many cardiac diseases can be diagnosed. ECG machines are safe and inexpensive. However, noise and other factors, which are called artifacts can produce spikes in ECG signals. These artifacts can be body movement of patients, electrode movement on a body, and power line interferences. Therefore, noise and artifacts must be removed from the ECG signals to ensure accurate ECG analyses. Different transforms are used for the preprocessing of ECG signals to remove noise and artifacts, and one of the most commonly used transform is the wavelet transform^6,7^. Several algorithms have been previously reported to detect P, QRS complex, and T waves, so as to realize noise and artifact-free ECG signals, and they have been validated over MIT-BIH arrhythmia database^8–13^. In^8^, a rapid-ramp effective algorithm was proposed for the detection of R peaks, which uses the slopes between adjacent signals to determine the occurrence of the R peaks. This algorithm is only applied to two records of the database and has higher-order complexity. In^9^, a combination algorithm based on empirical-mode-decomposition and the Hilbert transform was proposed to detect the R peaks in ECG signals. However, this algorithm is complicated and involves a large number of blocks for the detection of R peaks. Moreover, both of these algorithms are restricted to the detection R peaks only. Along with R peaks, to detect P and T peaks, Elgendi et al., proposed some algorithms based on two event-related moving averages (TERMA)^10–13^. These algorithms involve different building blocks such as filtering, enhancing, block-of-interest (BOI) generation for each peak, and thresholding. In these algorithms, the ECG signals are filtered using a Butterworth filter, and the output values are squared to enhance large values and minimize small values. After enhancement, window sizes are selected based on the duration and repetition intervals of the QRS wave. Next, BOI is generated for each peak using moving averages. The width of each block is calculated and compared with a threshold depending on the window size. Finally, the peaks are detected from each block. For the localization of P and T peaks, the samples before and after the detected R peaks, including the R peak samples, are set to zero depending on the RR interval. This algorithm provides acceptable results with regard to peak detection.

In this paper, to address the drawbacks of the above mentioned algorithms, based on the fusion of TERMA and fractional Fourier-transform (FrFT), we propose an algorithm that can produce better results. TERMA is used in economics to detect different events in trading, and moving averages are helpful in detecting the signals that contain specific events. Thus, these averages can also be used in ECG signals , which contain events such as P, QRS complex, and T waves. These waves repeat themselves after certain time intervals. Likewise, time-frequency analyses are relevant due to the large variations in P, QRS complex, and T waves. In this paper, we demonstrate how moving averages and time-frequency analyses can be exploited for the detection of these waves. Further, we showed that the proposed algorithm in this paper, has a significantly better performance than the existing algorithms.

Our second objective is to classify the CVD of a given ECG signal, if any. Classification involves two steps: feature extraction and classifier model selection. Many researchers have worked on the classification of ECG signals using the MIT-BIH arrhythmia database. Different preprocessing techniques, feature extraction methods, and classifiers have been used in previous studies and some of them are discussed in this paper. In^14^ features such as the R peak and RR interval were extracted using discrete-wavelet-transform (DWT), and multi-layer perceptron (MLP) was used in ECG classification. The obtained accuracy was $99.9\%$ but a total number of 301 features were used for classification. Similarly, in^15^, the R peak location and RR interval were extracted using db4 DWT, and to classify ECG signals, a feed-forward neural-network (FFNN) was trained with backpropagation. The sensitivity, specificity, and accuracy achieved by FFNN were $90\%, 90\%$, and $95\%$ respectively. In^16–20^ different classifiers such as Naive Bayes, Adaboost, support vector machines (SVM) and neural networks were used in classification.

### Our contribution

Our contributions are as follows:

- Our proposed FrFT-based algorithm exploits FrFT for the detection of P, QRS, and T waveform peaks. Additionally, it is simple and less complex than other algorithms, and it has outperformed the recently proposed TERMA algorithm in detecting P, QRS, and T peaks. The detection performance of the TERMA algorithm depends on CVD. In contrast, our proposed algorithm is more generic and outperforms TERMA for any CVDs.
- The second contribution is related to the CVD classification. The PR and RT durations calculated from the estimated locations of the P, R, and T peaks in the previous contribution are considered as features. Along with AR coefficients, these features significantly reduced the number of features required to classify CVD. We tried different features and improved the classification accuracy using MLP and SVM classifiers.
- Usually, the particular features chosen for a database do not necessarily perform well another database. We showed that PR and RT durations along with the age and sex features perform very well for different databases, and the computational complexity required was found to be significantly lower than that of state-of-the-art algorithms.
- We trained our model using MIT-BIH arrhythmia database^21^ and then tested it on two different databases, INCART^22^ and SPH^23^ respectively. The attained accuracies were $99.85\%$ and $68\%$.

**Figure 2:**
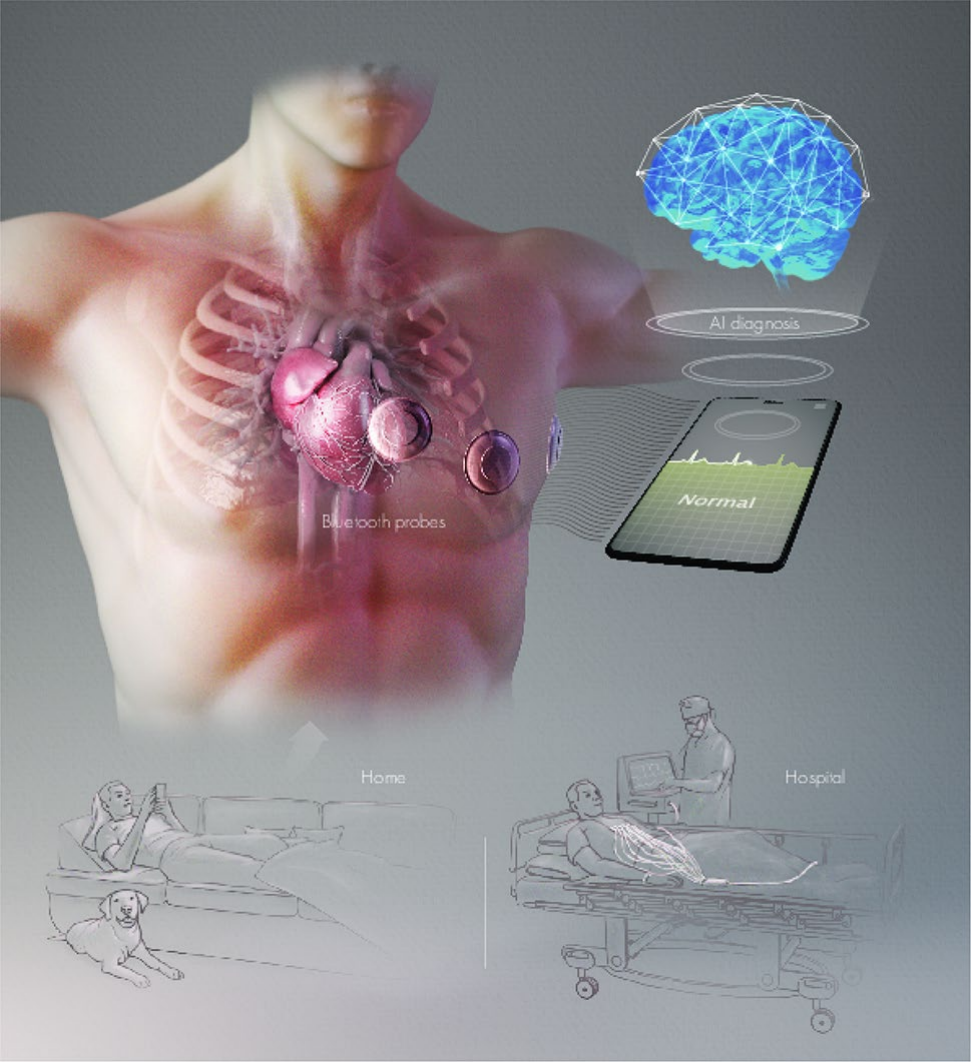
A cardiologist- and probe-less contemporary cardiovascular disease diagnosis system. Authors are thankful for the illustration created by Ivan Gromicho. Scientific Illustrator at Research Communication and Publication Services. Office of the Vice President for Research—King Abdullah University of Science and Technology.

The proposed algorithm can be used in futuristic cardiologist- and the probe-less systems as shown in Fig. 2. In such a system, probe-less ECG sensors are placed on the patient body and signals are transmitted with the help of Bluetooth to a processing device such as a mobile. The received signal can be processed and passed to a proposed machine learning algorithm for automatic CVD diagnosis.

### Paper organization

The rest of the paper is organized as follows. Section 2 describes the some techniques used in the proposed algorithm, and Sect. 3 describes the methodology used in peak detection in detail. Then, Sect. 4 describes the feature extraction and classification using machine learning and Sect. 5 presents the results of the proposed algorithm, which was validated over a variety of signals from two different databases. Finally, Sect. 6 concludes the paper.

## Some preliminaries

In this section, we discuss some important techniques that are used in the proposed methodology.

### Discrete wavelet transform

The approximate and detailed coefficients of DWT of a function *x*(*t*) are respectively defined as follows^24^:

$$W_{\phi }(j_o,k)=  \frac{1}{\sqrt{M}}\sum _{k=0}^{M-1}x(t)\phi _{j_o,k}(t)$$

and

1 $$ W_{\psi }(j,k)=  \frac{1}{\sqrt{M}}\sum _{k=0}^{M-1}x(t)\psi _{j,k}(t) , $$

where $j\ge j_o$, $j_o$ is the starting scale, $\phi _{j,k}(t)$ is the scaling function, and $\psi _{j,k}(t)$ is the wavelet function. The inverse discrete-wavelet-transform (IDWT) for given approximate and detailed coefficients is defined as follows:

$$\begin{aligned} x(t)=\frac{1}{\sqrt{M}}\sum _{j_o=0}^{J-1}W_{\phi }(j_o,k)\phi _{j_o,k}(t) +\frac{1}{\sqrt{M}}\sum _{j=j_o}^{J-1}W_{\psi }(j,k)\psi _{j,k}(t). \end{aligned}$$

### Moving averages

Moving averages result in smoothing out short-term events while highlighting long-term events. In trading, two moving averages are used together resulting in two crossovers. The use of these averages results in the detection of trading events. These averages can be used in the detection of P, QRS, and T waves. The implementation of the moving average results in higher numerical efficiency with less complexity. Therefore, the idea of using two moving averages is promising in analyzing biomedical signals.

### Fractional Fourier transform

The FrFT is the generic form of classical Fourier-transform with a parameter ($\alpha$) that shows order^25^. It was first introduced in mathematical literature years ago. FrFT is mainly used in solving the differential equations in quantum physics, but it can also be used in interpreting optics related problems. In recent years, the use of FrFT in optical applications has been increasing. Many new applications have been proposed in the field of data processing of signals because of the useful characteristics of FrFT in the time-frequency plane. The FrFT of a signal can be defined as follows^26^:

$$\begin{aligned} {\text {FrFT}}^{\phi }(t,u) = F^{\alpha }(x(t)) = X_{\phi }(u)=\int _{-\infty }^{\infty }x(t)K_{\phi }(t,u)dt \end{aligned}$$

where $\alpha$ is the order of FrFT and $\phi =\alpha \pi /2$ is the angle of rotation. While $F^{\alpha }(\cdot )$ denotes the FrFT operator and $K_{\phi }(t,u)$ represents the kernel of FrFT and is defined as

$$\begin{aligned} K_{\phi }(t,u)= {\left\{ \begin{array}{ll} {\sqrt{\frac{1-j\cot {\phi }}{2\pi }}}\exp (j {\frac{t^2+u^2}{2}}\cot {\phi }-j t u \csc {\phi }),\phi \ne n\pi \\ \delta (t-u), \quad {\text {for}} \quad \phi =2 n \pi \\ \delta (t+u), \quad {\text {for}} \quad \phi =2(n+\frac{1}{2})\pi ,\\ \end{array}\right. } \end{aligned}$$

where *n* is an integer.

## Proposed methodology

**Figure 3:**
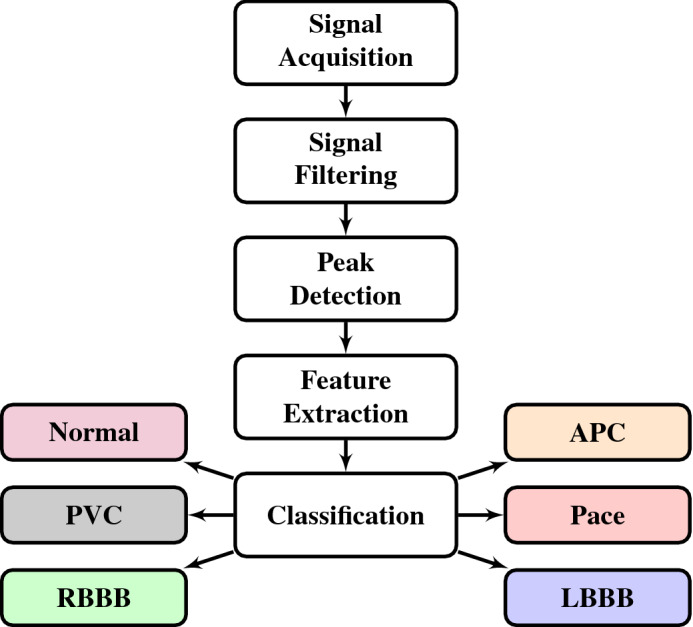
Block diagram of the proposed methodology, [ PVC: Premature ventricular contraction, RBBB: Right bundle branch block, APC: Atrial premature contraction, LBBB: Left bundle branch block].

As motioned earlier, for the accurate detection of P, QRS, and T waves, artifacts and noise should be removed from signals. Figure 3 shows the block diagram of the proposed three-step methodology. (1) To remove noise and artifacts, the conventional wavelet-transform-based filtering method is used, (2) for the detection of P, QRS complex, and T waveforms TERMA and FrFT are fused together to improve the detection performance, and (3) machine learning algorithms are applied to classify ECG signals to determine the CVD if any. The individual tasks are discussed in detail in the following subsections.

### Signal filtering

The ECG signals are non-stationary, i.e., their frequency response changes with respect to time. Similarly, the noise and artifacts contaminating the ECG signal are non-linear, and their probability-distribution function is time-dependent. Conventional Fourier transform techniques do not provide time localization, while DWT provides time localization. Therefore, DWT can better deal with non-stationary signals. First step is to remove the baseline drift using DWT^27^. For this purpose, first of all, the central frequency, $F_c$, (also called $F_c$ factor) is calculated for the wavelet, which ranges from 0 to 1 depending on the similarity between the signal and chosen wavelet.

For the ECG signals, Daubichie-4 (db4) has the highest $F_c$ factor, which is approximately equal to 0.7. Next, pseudo-frequency, $F_a$, is calculated at each scale using the expression^27^

2 $$\begin{aligned} F_{a}=\frac{F_c F_s}{2^{a}}, \end{aligned}$$

where *a* and $F_s$ represent the scale and sampling frequency of the ECG signals, respectively. The baseline drift is mostly localized around 0.5Hz^28^. For the MIT-BIH $F_s = 360$, therefore using (2), the scales corresponding to different pseudo frequencies can be easily calculated. Decomposition should be up to scale 9 that corresponds to $F_a=0.5$. Therefore, the ECG signal is decomposed into approximation and detailed coefficients using the db4 wavelet up to scale 9. The approximate coefficients corresponding to the baseline drift are removed, and the signal is reconstructed using IDWT to obtain a baseline drift-free signal^29^.

Once the baseline drift-free signal is obtained, the next step is to remove high frequency noise. It was reported in^30^, that most of the QRS complex energy is concentrated within the range of 8 to 20 Hz. The SNR has been calculated at different levels, which shows that decomposition up to level 6 is required to capture the QRS complex wave. Therefore, the signal is reconstructed using the detailed coefficients of levels 4, 5, 6 and the approximation coefficients of level 6.

**Figure 4:**
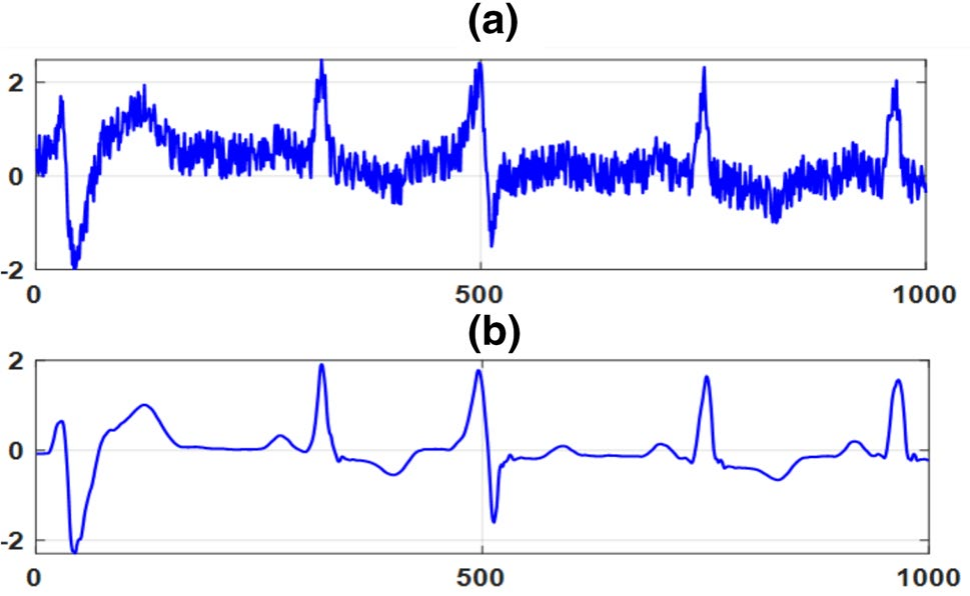
(**a**) ECG signal with the baseline drift and high frequency noise. (**b**) The baseline drift and high frequency noise free signal after DWT based filtering.

The detailed coefficients of levels 1, 2 and 3 contain high frequencies ranging from 50 Hz to 100 kHz. These frequencies belong to muscle contraction noise. Therefore, at these levels, the details are discarded, and the approximations are retained to remove high-frequency noise. The resulted signal has highest SNR because the high frequency detailed coefficients are discarded. Figure 4 shows the baseline drift and high frequency noise-free signal. In the TERMA algorithm, to detect peaks, the artifact and noise free signal is squared to enhance the peak values, a BOI is generated for each wave, and thresholding is finally applied. In the following subsection, we showed how the TERMA algorithm detection performance can be improved by exploiting FrFT.

### R peak detection using the fusion algorithm

**Figure 5:**
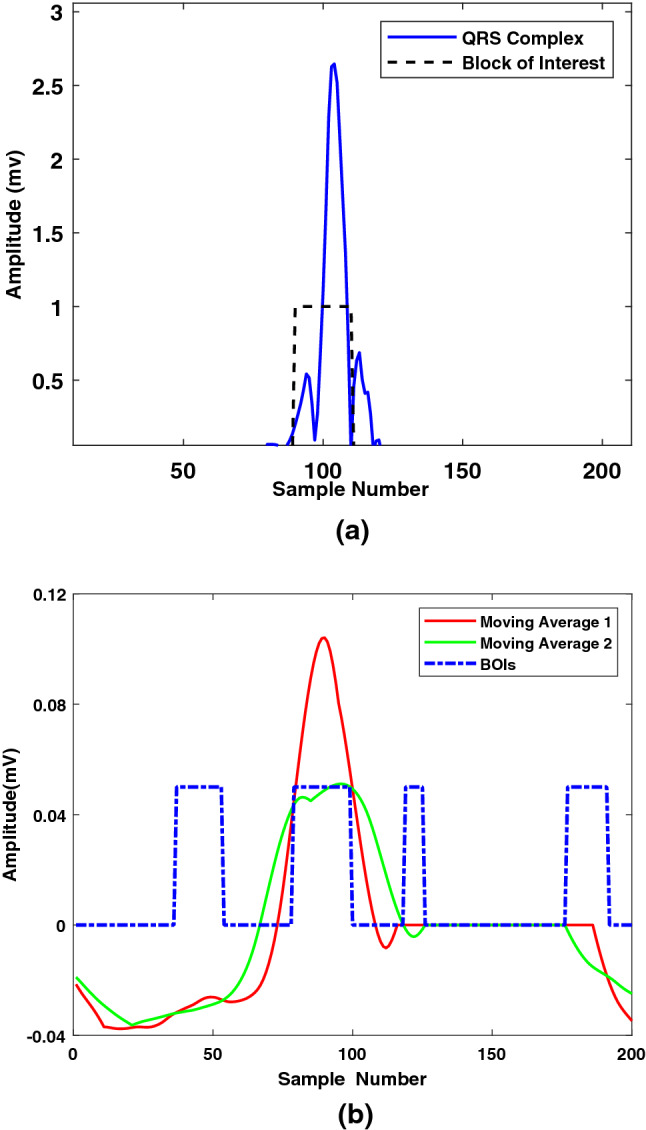
(**a**) Block of interests generation for the detection of R peaks. (**b**) Block of interests generation for the detection of P and T peaks.

**Figure 6:**
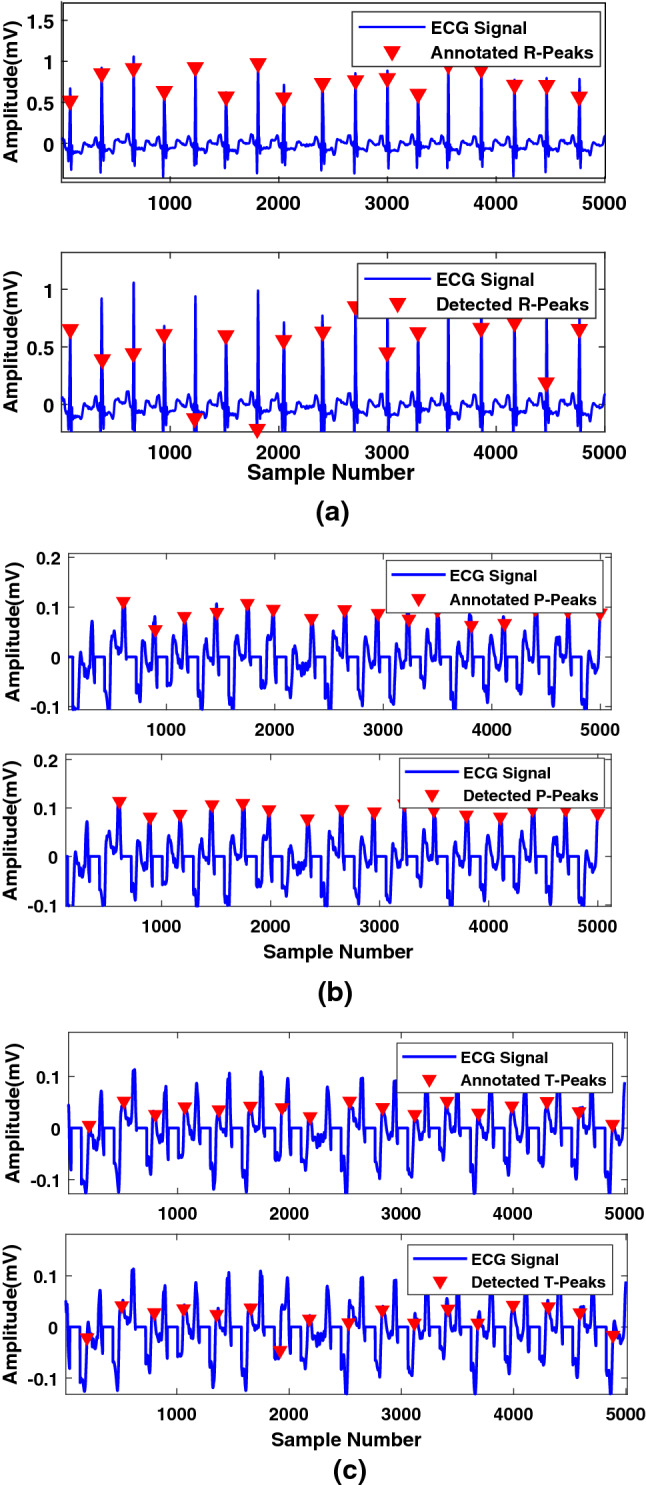
(**a**) Actual annotations for the R-peak in ECG record 200 m, (**b**) Actual annotations for the P-peak in ECG record 103 m, and (**c**) Actual annotations for the T-peak in ECG record 103m and the detected T-peaks after applying the algorithm.

In the ECG signal, the maximum change in frequency occurred at the R peak. By taking the Fourier transform of the ECG signal, the time localization can be lost. Therefore, in this step, FrFT was applied to the noise-free signal to rotate the signal in the time-frequency plane^31^. As seen in the preliminaries, the FrFT operation comprises a chirp multiplication, followed by a chirp convolution, and lastly another chirp multiplication. Rotating the signal with a higher value of $\alpha$ is like moving closer to the frequency domain of the signal, while rotating it with a lower value of $\alpha$ is like moving toward the time domain of the signal. In R-peak detection, time localization is very important^32^. Using the hit and trial method, we found that the value of $\alpha = 0.01$ appropriately enhances R-peaks and makes them easy to detect. After applying FrFT, the R peak was more enhanced by squaring each sample. After the enhancement, two moving averages based on event and cycle were calculated as follows:

$$\begin{aligned} {\text {MA}}_{event}(n)= & \, \frac{1}{W_1} \sum _{k=-l}^l x(n+k),\\ {\text {MA}}_{cycle}(n)= & \, \frac{1}{W_2} \sum _{k=-p}^p x(n+p), \end{aligned}$$

where $W_1$ depends on the duration of the QRS complex, and $W_2$ depends on the heartbeat duration. The mean ($\mu$), of the enhanced signal is calculated and multiplied by a factor ($\beta$) whose optimum value was chosen by hit and trial method. The output number is denoted by $\gamma = \beta \mu$, and was added to ${\text {MA}}_{cyclic}$ to generate threshold values. Each value of the ${\text {MA}}_{event}$ was compared with the corresponding threshold value. If ${\text {MA}}_{event}(n)$ was greater than the *n*th threshold, one is assigned. Otherwise, zero is assigned in a new vector. This way, a train of nonuniform rectangular pulses is generated. Finally, the pulses that have widths equivalent to $W_1$ are the blocks that contain the desired event as shown in Fig. 5a. In each block, the maximum value in the corresponding enhanced signal is considered an R peak value. This process is explained in detail in^12^. Figure 6a shows that the R peaks were accurately detected after applying the proposed algorithm.

### P and T peak detection using the fusion algorithm

To detect P and T peaks, TERMA uses a complicated threshold. We reduced the overall computation complexity of the algorithm by applying a simplified threshold. The first step of the algorithm is to remove the R peaks to make the P and T peaks prominent. Thus, 30 samples (0.083 s) before the R peak and 60 samples (0.166 s) after the R peak were set to 0 in the noise-free signal. In the chosen interval, the expectation of the P and T waves was almost zero for any CVD. After the QRS interval removal, the signal was rotated in time-frequency plane using FrFT to enhance the P and T peaks. Similar, to the previous section, block of interests were generated as shown in Fig. 5b, using two moving averages defined as follows:

$$\begin{aligned} {\text {MA}}_{peak}(n)= & \, \frac{1}{W_3} \sum _{k=-q}^q x(n+q)\\ {\text {MA}}_{wave}(n)= & \, \frac{1}{W_4} \sum _{k=-r}^r x(n+r), \end{aligned}$$

where $W_3$ depends on the P wave duration, $W_4$ depends on the QT interval, $q={\frac{W_3-1}{2}}$, and $r = {\frac{W_4-1}{2}}$. For a normal healthy person, the P wave duration can be $(100\pm 20)$ ms, whereas the QT interval can be $(400 \pm 40)$ ms. To detect P waves, instead of a normal size, a smaller window was chosen to consider the special cases of arrhythmias. Here, in contrast to the case of the R-peak detection, the threshold values were simply the values of the second moving average. If the first moving average was greater than the corresponding second moving average one is assigned. Otherwise, zero is assigned in a new vector. This way, a train of nonuniform rectangular pulses is generated.

Finally, a threshold based on the PR, RR and RT intervals was applied to distinguish the generated blocks from the blocks that contain P and T peaks. If the distance between the maximum value of the block and the nearest R peak is within the predefined PR interval, the maximum value of the block is referred to as the P peak. If the distance between the maximum value of the block and the nearest R peak is within the predefined RT interval, the maximum value of the block is referred to as the T peak. Figure 6b and c shows that the P and T peaks were accurately detected after applying the proposed algorithm. In this work, similar to the TERMA algorithm, we have detected normal and merged T peaks. Therefore, there is a need to investigate T peaks with different shapes such as inverted, biphasic negative-positive, and biphasic positive-negative. Moreover, different types of moving averages can help in further analysis of ECG signals. This algorithm is not designed to work for the additional U wave after the T peak. These aspects would be investigated in our future work.

## Classification of The ECG signal

In this section, to classify the given ECG signal according to CVD, machine learning was applied. In machine learning, training datasets with corresponding labels are fed in an algorithm, where different features are extracted from each dataset and a model is formed to predict test data labels. This is called supervised machine learning. It helps in the automatic decision-making process by building different models from sample data. Data training includes two steps, feature extraction and classification, as discussed in the following subsections.

### Feature extraction

Different features can be extracted from the ECG signal. For example, the estimation of different peaks can be used to find the time intervals between different peaks. Since these time intervals represent different cardiac conditions, they can be considered as features. Moreover, auto-regressive (AR) model coefficients of the ECG signal can be used as a feature^33^. The AR model of order *p*, AR(*p*), can be defined as follows:

3 $$\begin{aligned} x(n)= \sum _{i=1}^{p}a(i)x(n-i)+e(n), \end{aligned}$$

where *a*(*i*) is the $i\hbox {th}$ coefficient of AR model, *e*(*n*) is a white noise with a zero mean, and *p* is the order. The optimum order of the AR model depends on the number of factors. Higher order AR model yields more accurate modeled signals but at the cost of higher computational complexity in calculating the coefficient values. Similarly, other features, such as the wavelet transform coefficients, mean, variance, age, sex, and cumulant, can be extracted to classify the CVD of the ECG signal. Feature extraction is very important because it shows which type of inputs can better represent the signal. In this work, MIT-BIH arrhythmia^21^ and SPH^34^ database signals were used.

### Feature matrix

The feature matrix contains feature information of ECG beats taken from different records of the arrhythmia database. Each row of the matrix shows the feature information of a single heartbeat. Each row includes different features of heartbeats taken from the datasets. For example, if we take four coefficients from the AR model, *n* coefficients from the FrFT of the given heartbeats, and two intervals PR and RT as features, the feature vector can be written as follows: $\{a_1, a_2, a_3, a_4, f_1, f_2, \ldots ,f_n, PR, RT\}$. The feature matrix can be formed with such multiple rows.

### Supervised machine learning algorithms

The classification of the ECG signal is a very important and challenging task. It can provide substantial information about the CVDs of a patient without the involvement of a cardiologist. Only a technician is required to attach the probes, and the machine learning based solution can automatically diagnose the CVDs of the patient. This technique can immediately prioritize the patients that need urgent medical attention^35^. In this work, the SVM and MLP supervised learning algorithms were used for classification and they were briefly discussed in the following subsections.

#### Support vector machine classifier

The SVM algorithm can be used in classification and regression problems^36^. In SVM, data is plotted in an *l*- dimensional space, where *l* denotes the number of features. After plotting the data, classification is performed by finding a hyperplane that differentiates between different classes. The maximization of the margin optimizes the hyperplane. Then, the hyperplane, that is at a higher distance from the closest data points among other hyperplanes, is chosen. The SVM solves the following quadratic problem:

4 $$\begin{aligned}&\max _{\alpha \ge 0} \left( \sum _{i=1}^{l}\alpha _{i} - \frac{1}{2}\sum _{i,j=1}^{l}\alpha _{i}\alpha _{j}y_{i}y_{j}K(X_{i}, X_{j})\right) \end{aligned}$$

5 $$\begin{aligned}&{\text{ subject }} {\text{ to }} \qquad \sum _{i=1}^{l}\alpha _{i}y_{i}=0 \end{aligned}$$

6 $$\begin{aligned}&\alpha _{i}\le C, i=1,2,\ldots ,l, \end{aligned}$$

where $X_i$, $X_j$ are input features, $y_i$, $y_j$ are class labels , $\alpha _i\ge 0$ are Lagrangian multipliers, *C* is a constant, and K($X,X_1$) is a kernel function^37^. A very common kernel function is the Gaussian radial basis function:

7 $$\begin{aligned} K(X,X_{1})=\exp {-\frac{{\Vert {X-X_1} \Vert }^2}{2\sigma ^{2}}}. \end{aligned}$$

The SVM is very effective in higher dimensional spaces and when the number of dimensions is greater than the number of samples.

#### Multi-layer perceptron classifier

Artificial-neural-network (ANN) algorithms classify regions-of-interest using a methodology that performs functions similar to those of the human brain, such as understanding, learning, solving problems, and making decisions. The ANN architecture consists of three layers. The first layer is the input layer, and the input parameters determine the number of neurons in this layer. The last layer is the output layer, and the number of neurons in this layer represents the number of output classes. The layers between the input and output layers are called the hidden layers^38^. MLP was used in this work, and it is a subclass of the feed-forward ANN.

## Simulation results and discussion

This section is divided into three parts, which are dedicated respectively to peak detection, classification, and cross-database training and testing.

### Detection of ECG peaks

In the first part of the simulation, using our proposed FrFT-based algorithm, the P, R, and T peaks are detected, and the proposed algorithm is validated over all the 48 records of the MIT-BIH database. Lead II (MLII) data is used in this paper. Our algorithm works independent of the amplitude of the waveform, so any lead data can be used for the peak detection. Moreover, the performance is assessed using different metrics reported in the literature, such as sensitivity, positive predictivity, and error-rate, which are defined as follows^39,40^:

$$\begin{aligned} \hbox {Sensitivity (SE)}= & \, \frac{{\text {TP}}}{{\text {TP}}+{\text {FN}}}, \\ \hbox {Positive Predictivity (+Pr)}= & \, \frac{{\text {TP}}}{{\text {TP}}+{\text {FP}}},\\ \hbox {Error Rate (Err) }= & \,\, \frac{{\text {FP}}+{\text {FN}}}{{\text {TP}}}, \end{aligned}$$

where TP denotes the true-positive, FN denotes the false-negative defined as the annotated peaks not detected by the algorithm, and FP denotes the false-positive defined as the peaks detected by the algorithm but not actually present. If a peak is detected within the 30 ms interval of the annotated peak, it is defined as TP. To assess the performance of the algorithm, we observed TP, FN, and FPs. In Table 1, the R peak detection performance of our proposed algorithm is compared with the TERMA algorithm. Both algorithms were tested over the 48 records of the MIT-BIH arrhythmia database. As seen, the proposed algorithm performed slightly better than the TERMA algorithm.

**Table 1:** Performance comparison of the R-peak detection for the 48 records of the MIT-BIH database.

Algorithms	SE (%)	+Pr (%)	Err
Proposed algorithm	99.83	99.90	0.00259
TERMA^12^	99.78	99.87	0.00298

Similarly, the detection performance of the proposed algorithm in the detection of P and T waves was compared with that of TERMA algorithm as shown in Table 2. In Table, we compared the reported performance of TERMA algorithm in^13^, where only 10 records of MIT-BIH database were selected. It can be seen that our proposed algorithm outperforms TERMA algorithm.

**Table 2:** Performance comparison of P and T-peak detection for the first 10 and remaining 38 records of the MIT-BIH database.

10 Records	Detection of P Peaks		Detection of T Peaks	
Algorithms	SE (%)	+Pr (%)	SE (%)	+Pr(%)
Proposed algorithm	99.28	99.05	99.94	99.83
TERMA^13^	98.05	97.11	99.86	99.65
**38 Records**				
Proposed algorithm	75.8	0.40	67.5	0.51
TERMA^13^	59.2	1.04	42.8	1.15

In Table 2, both algorithms were also tested on the remaining 38 records of the MIT-BIH database. Here, significant difference can be seen in the detection performance of both algorithms. For the P peak detection, our proposed algorithm resulted in SE of an $75.8\%$ and an Err of 0.40 compared with an SE of $67.5\%$ and Err of 0.51 in the case of TERMA. For the T peaks detection, proposed algorithm results in SE of $59.2\%$ and Err of 1.04 compared with an SE of $42.8\%$ and Err of 1.15 in the case of the TERMA algorithm as shown in the table. This shows that the detection performance of the TERMA algorithm is limited to a few CVDs, while our proposed algorithm performs very well for the other CVDs in the MIT-BIH database.

Overall, it was found that our proposed algorithm performs better than the TERMA algorithm and other previously presented algorithms.

### Classification of CVDs

In the second part of the simulation, we classify the ECG signals according to their CVDs. Here, for all simulations 70% of the feature data was allocated to train the machine learning model while 30% was kept for testing^37^. Therefore, different features were extracted from the signals for the classification. Then, the extracted features were passed into the SVM and MLP classifiers to classify the input ECG signals as normal, PVC, APC, LBBB, RBBB, and PACE heartbeats. To compare the performance of the proposed classifier with that of the existing ones, the following performance metrics were used:

$$\begin{aligned} \hbox {Overall Accuracy}= & \, \frac{{\text {TP}}+{\text {TN}}}{{\text {TP}}+{\text {TN}}+{\text {FP}}+{\text {FN}}} ,\\ \hbox {Precision}= & \, \frac{{\text {TP}}}{{\text {TP}}+{\text {FN}}}, \\ \hbox {Recall}= & \, \frac{{\text {TP}}}{{\text {TP}}+{\text {FP}}},\\ f_{1}\hbox {-Score}= & \, 2.\frac{\hbox {Precision }\times \hbox { Recall}}{\hbox {Precision }+\hbox { Recall}}, \end{aligned}$$

where TN denotes a true-negative, which is defined as, the patient has a CVD and the classifier also predicts that the patient is not normal.

As we know, the MIT-BIH database contains limited ECG signals from only 48 patients. For machine learning algorithms, the quantity of data is crucial. Therefore, for classification, we tested the proposed algorithms on the recently reported Shaoxing SPH database^23^. This database contains 12 lead ECG signals from 10,646 patients. In contrast to the MIT-BIH ECG signal sampling rate of 360 samples/s, the sampling rate of the SPH ECG signal is 500 samples/s. The data set consists of four folders containing ECG raw data, ECG denoised data, diagnosis data, and attributes. This database consists of 11 common rhythms and 67 additional cardiovascular conditions. Each of the 12 lead signals is 10 s long i.e., 5000 samples for each lead. In this database, 11 rhythms are merged into four groups SB, AFIB, GSVT, and SR. The SB group only includes sinus bradycardia, the AFIB group consists of atrial fibrillation and atrial flutter (AF), the GVST group contains supra ventricular tachycardia, atrial tachycardia, atrioventricular node reentrant tachycardia, atrioventricular reentrant tachycardia, and sinus atrium to the atrial wandering rhythm, while the last SR group includes sinus rhythm and sinus irregularity.

For the first classification-simulation, the extracted features were passed to the SVM classifier. The parameter values of *C* and $\gamma = \frac{1}{2\sigma ^2}$ were respectively adjusted to 65536 and $2.44\times 10^{-4}$^37^. The scikit-learn library of Python was used for machine learning model building^41^. In^37^, to classify an ECG signal, 36 features are extracted from it, where 32 features were the DWT (db4) of the signal and 4 were the coefficients of AR model. However, in the proposed classifier, a feature matrix was generated using only four features, where two features were extracted using the estimated P, R, and, T peaks, which are PR and RT intervals, whereas the other two were age and sex. Both classifiers were trained and tested on the records of the MIT-BIH and SPH databases. In the case of MIT-BIH database, the number of heartbeats extracted from the Normal, LBBB, RBBB, PACE, PVC, and APC records was 2237, 2490, 2165, 2077, 992, and 1382 respectively. However, in the case of SPH, the features were extracted from all heartbeats of 10,646 patients. The corresponding performances of both classifiers for the MIT-BIH and SPH databases is shown in Table 3. In the case of the MIT-BIH database, the overall accuracy of the classifier proposed in^37^ with 36 features was 99.6%. However, in the case of the SPH database, it significantly decreased to 37.1%. Nevertheless, in the case of the MIT-BIH database, the accuracy of our proposed classifier with only four features was 82.2%, but it became 84.2% in case of the SPH database, so it is much better and more stable than that of the proposed classifier in^37^. The computational complexity comparison of the feature extraction for both classifiers is also shown in the Table 3. The computational complexity to find the AR coefficients is ${\mathcal{O}}(p^3) + {\mathcal{O}}(p^2N)$, and DWT is ${\mathcal{O}}(LN)$, and $\alpha$ shows the computational complexity of finding the R peaks, where *L* is the number of decomposition levels and *N* is the number of samples in one heartbeat. In our algorithm, to find the R peak using FrFT, the computational complexity was ${\mathcal{O}}(N\log _2N)$. In^37^, instead of estimations, annotated R peaks were used, so there were some computation cost denoted by $\eta$ depending on the used algorithm. Considering the same computational complexity for estimating R peaks, the computational complexity of our proposed classifier is lower by an order of ${\mathcal{O}}(p^3) + {\mathcal{O}}(p^2N)$, which is the computational cost of AR model. In the table, by adding a few other features, the corresponding accuracy and computational complexity were also shown. It can be seen in terms of computational complexity and accuracy, PR, RT, age, and sex are the most promising ones for different databases.

**Table 3:** The SVM and MLP classifier overall accuracy and computational complexity for the MIT-BIH and SPH databases using different sets of features.

No of features	Extracted features	Accuracy MIT-BIH		Accuracy SPH		Feature extraction complexity
		SVM (%)	MLP (%)	SVM (%)	MLP (%)	
36	AR+DWT^37^	99.6	99.8	37.1	38.2	${\mathcal{O}}(p^3) + {\mathcal{O}}(p^2N) + {\mathcal{O}}(LN)+ \eta$
4	AR	92.6	93	37	39	${\mathcal{O}}(p^3) + {\mathcal{O}}(p^2N)$
4	PR+RT+Age+Sex	82.2	80	84.2	90.7	${\mathcal{O}}(N\log _2N) + {\mathcal{O}}(LN)$
6	AR+PR+RT	96	94	82.96	91.17	${\mathcal{O}}(p^3)+ {\mathcal{O}}(p^2N) + {\mathcal{O}}(N\log _2N) + {\mathcal{O}}(LN)$
8	AR+PR+RT+Age+Sex	98.4	83	83.87	87.6	${\mathcal{O}}(p^3)+ {\mathcal{O}}(p^2N) + {\mathcal{O}}(N\log _2N) ++ {\mathcal{O}}(LN)$
40	AR+DWT+PR+RT+Age+Sex	99.8	96.5	82.7	65	${\mathcal{O}}(p^3) +{\mathcal{O}}(p^2N) + {\mathcal{O}}(N\log _2N) + {\mathcal{O}}(LN)$

In the second simulation, the first simulation steps were repeated with the MLP classifier. The corresponding simulation results are also shown in Table 3. Here again, it can be seen that in the case of the MIT-BIH database, the MLP classifier’s accuracy with 36 features was 99.8%, but in the case of SPH, it decreased to 38.2%. However, with our proposed 4 features, in the case of the MIT-BIH database, the accuracy was 80% while in the case of the SPH database, it was 90.7%. Therefore, we can say that our proposed classifier has more stability with respect to database changes than other classifiers.

Table 4, shows a performance comparison of SVM and MLP for the MIT-BIH and SPH databases in terms of precision, recall, and $F_1$-Score for individual CVDs. In the table, it can be seen that MLP performed much better than SVM on the SPH database. While, for some diseases, the performance of the SVM classifier was slightly better than that of MLP in the case of the MIT-BIH database. Therefore, we can say that MLP is a better choice for both databases. The confusion matrix for the MIT-BIH using MLP classifier is shown in Table 5. The confusion matrix for other classifiers can be easily calculated.

### Cross database training and testing

In the third part of the simulation, the MLP classifier was trained using the MIT-BIH arrhythmia database and then tested on the St. Petersburg INCART^22^ and SPH^23^ databases to classify the Normal, RBBB, and PVC heartbeats. All three databases have different sampling rates. Therefore, all the signals were resampled to a frequency of 128 Hz for the simplicity. The data extracted from these databases was already baseline wander and noise free, so there was no need of preprocessing. Different features, such as age, sex, PR, and RT intervals were extracted. The overall accuracy of the trained model on the INCART database and SPH database was $99.85\%$ and $68\%$ respectively. The detailed performance of the classifier for various CVDs in terms of precision, recall, and $F_1-$Score is shown in Table 6.

**Table 4:** The performance comparison of the SVM and MLP classifiers for the MIT-BIH and SPH databases using the features PR, RT, age, and sex.

MIT-BIH	SVM			MLP		
Diseases	Precision	Recall	f1-Score	Precision	Recall	f1-Score
Normal	0.524	0.985	0.684	0.815	0.746	0.779
LBBB	1.000	1.000	1.000	0.681	0.871	0.765
RBBB	0.818	0.070	0.130	0.749	0.746	0.748
PACE	1.000	1.000	1.000	0.868	0.833	0.851
PVC	1.000	1.000	1.000	1.000	0.844	0.916
APC	1.000	1.000	1.000	0.845	0.748	0.793
**SPH**						
AFIB	0.666	0.580	0.620	0.883	0.782	0.830
SB	0.746	0.818	0.780	0.879	0.898	0.889
SR	0.957	0.992	0.974	0.941	0.988	0.964
GSVT	0.842	0.818	0.830	0.895	0.899	0.897

In the case of the SPH database, as shown in the Table 6, classifier was unable to correctly classify the RBBB and PVC heartbeats, because our proposed algorithm was unable to detect inverted ,biphasic negative-positive and biphasic positive-negative T peaks, which may present in RBBB and PVC. It results in degradation of the overall classifier accuracy. There is a drawback associated with cross database processing. The classifier works only when disease features are normalized and normal patient features are not normalized for both training and testing. If we apply normalization to all the training and testing data, the accuracy of the classifier further degrades. However, this condition is not realistic and needs further investigation.

**Table 5:**
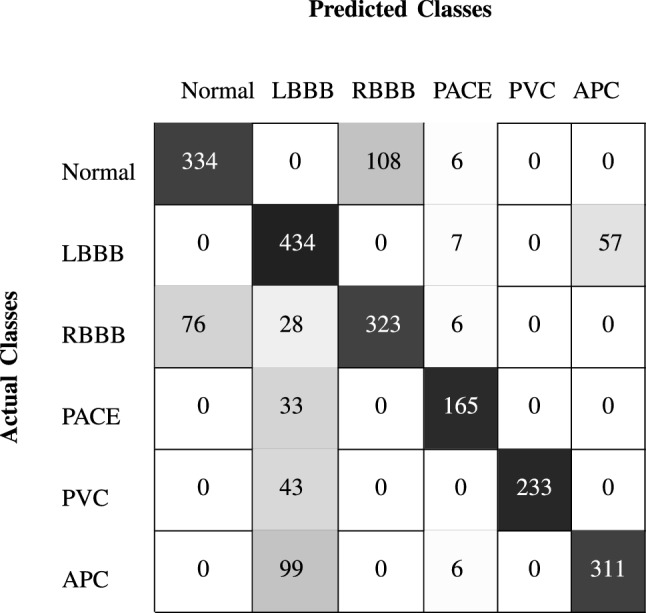
Confusion matrix for MIT-BIH database using MLP classifier.

In the future, we plan to work on this problem to further increase the overall prediction accuracy.

**Table 6:** Performance of cross-database processing of the INCART and SPH databases.

Diseases	INCART			SPH		
	Precision	Recall	f1-Score	Precision	Recall	f1-Score
Normal	1.000	0.996	0.998	1.000	0.990	0.995
RBBB	1.000	1.000	1.000	0.727	0.080	0.144
PVC	0.995	1.000	0.997	0.511	0.970	0.669
Macro average	0.999	0.999	0.999	0.746	0.680	0.603
Weighted average	0.999	0.999	0.999	0.746	0.680	0.603

## Conclusion

In this work, a fusion algorithm based on FrFT and TERMA was proposed to detect R, P, and T peaks. Conventional wavelet transform method were used to denoise signals, whereas the use of FrFT in the TERMA algorithm significantly improved the peak detection performance. We applied the proposed peak detection algorithm in the MIT-BIH arrhythmia database, and it performed slightly better than the TERMA algorithm in the detection of the R peak, while significantly better than it in the detection of the P and T waveforms. Moreover, in contrast to the TERMA algorithm, the performance was independent of CVDs. After the peak detection, the results were used to find the PR and RT intervals as two features of the ECG signal for the classification. We used two classifiers with different features and found that MLP performs better than SVM for a variety of ECG signals. Both classifiers were tested on the two databases. Finally, we designed a classifier for cross-database training and testing. This is a challenging task, and as far as we know, there have not been any available works in this direction. Our initial results are promising and to further improve the results, will be our future work.

A demo of the work can be seen at the link https://www.youtube.com/watch?v=3tfin4sSBFQ. In the demo video, the algorithm is explained in the first part, while in the second part initial wireless ECG diagnosis system is presented. The ECG signal from the AD8232 ECG module is transmitted with the help of Arduino and Bluetooth transmitter and received by the Bluetooth receiver of an android mobile phone that run an Android app to display the signal on the mobile screen. In the initial version only raw signal display is included in the Android app, the algorithms proposed in this paper will be included in the developed Android app in the ongoing work.

## References

[1] Dagenais GR, Leong DP, Rangarajan S, Lanas F, Lopez-Jaramillo P, Gupta R, Diaz R, Avezum A, Oliveira GBF, Wielgosz A (2020). Variations in common diseases, hospital admissions, and deaths in middle-aged adults in 21 countries from five continents (PURE): A prospective cohort study. Lancet.

[2] Rajni IK (2013). Electrocardiogram signal analysis—An overview. Int. J. Comput. Appl..

[3] Clifford, G. D., Azuaje, F. & McSharry, P. Advanced methods and tools for ECG data analysis. *Artech* (2006).

[4] Malmivuo, J. & Plonsey, R. *Bioelectromagnetism: Principles and Applications of Bioelectric and Biomagnetic fields* (Oxford University Press, 1995).

[5] Moody GB, Mark RG (2001). The impact of the MIT-BIH arrhythmia database. IEEE Eng. Med. Biol. Mag..

[6] Misiti, M. Inc MathWorks, Wavelet Toolbox for use with MATLAB. *Math Works* (1996).

[7] Thiamchoo, N. & Phukpattaranont, P. Application of wavelet transform and shannon energy on R peak detection algorithm. In *International Conference on Electrical Engineering/Electronics, Computer, Telecommunications and Information Technology (ECTI-CON)*, pp. 1–5 (2016).

[8] Adeluyi , O. & Lee, J. A. R-reader: A lightweight algorithm for rapid detection of ECG signal R-peaks. In *IEEE International Conference on Engineering and Industries (ICEI)*, pp. 1–5 (2011).

[9] Mabrouki, R., Khaddoumi, B. & Sayadi, M. R peak detection in electrocardiogram signal based on a combination between empirical mode decomposition and Hilbert transform. In *IEEE International Conference on Advanced Technologies for Signal and Image Processing (ATSIP)*, pp. 183–187 (2014).

[10] Elgendi, M., Jonkman, M. & De Boer, F. R wave detection using coiflets wavelets. In *IEEE 35th Annual Northeast Bioengineering Conference*, pp. 1–2 (2009).

[11] Elgendi M (2013). Fast QRS detection with an optimized knowledge-based method: Evaluation on 11 standard ECG databases. PLOS ONE.

[12] Elgendi M (2016). Terma framework for Biomedical signal analysis: An economic-inspired approach. Biosensors.

[13] Elgendi M, Meo M, Abbott D (2016). A proof-of-concept study: Simple and effective detection of P and T waves in arrhythmic ECG signals. Bioengineering.

[14] Ayub S, Saini J (2011). ECG classification and abnormality detection using cascade forward neural network. Int. J. Eng. Sci. Technol..

[15] Naima, F. & Timemy, A. Neural network based classification of myocardial infarction: A comparative study of Wavelet and Fourier transforms. *BoD-Books on Demand* (2009).

[16] Padmavathi S, Ramanujam E (2015). Naïve Bayes classifier for ECG abnormalities using multivariate maximal time series motif. Procedia Comput. Sci..

[17] Rajesh KN, Dhuli R (2018). Classification of imbalanced ECG beats using resampling techniques and Adaboost ensemble classifier. Biomed. Signal Process. Control.

[18] Maciejewski, M. & Dzida, G. ECG parameter extraction and classification in noisy signals. In *Signal Processing: Algorithms, Architectures, Arrangements, and Applications (SPA)*. IEEE. 2017, 243–248 (2017).

[19] Kaistha T, Mahajan A, Ahuja K (2016). A novel approach for extraction and classification of ECG signal using SVM. Int. J. Comput. Technol. Appl..

[20] Xiong, Z., Stiles, M. K. & Zhao, J. Robust ECG signal classification for detection of atrial fibrillation using a novel neural network. In *Computing in Cardiology (CinC)*. IEEE, 2017, 1–4 (2017).

[21] George, M., & Roger, M. *MIT-BIH arrhythmia database*. https://www.physionet.org/content/mitdb/1.0.0/.

[22] Sharma, N.: *ECG Lead-2 data set PhysioNet (Open Access)*. https://www.kaggle.com/nelsonsharma/ecg-lead-2-dataset-physionet-open-access.

[23] Zheng J, Zhang J, Danioko S, Yao H, Guo H, Rakovski C (2020). A 12-lead electrocardiogram database for arrhythmia research covering more than 10,000 patients. Sci. Data.

[24] Furht, B. (ed.) p. 188, Springer US, Boston, MA (2008).

[25] Ozaktas HM, Arikan O, Kutay MA, Bozdagt G (1996). Digital computation of the fractional Fourier transform. IEEE Trans. Signal Process..

[26] Sejdić E, Djurović I, Stanković L (2011). Fractional Fourier transform as a signal processing tool: An overview of recent developments. Signal Process..

[27] Subramaniam, S. R., Ling, B. W. K., Georgakis, A. Motion artifact suppression in the ECG signal by successive modifications in frequency and time. In *IEEE Annual International Conference of the IEEE Engineering in Medicine and Biology Society (EMBC)*, pp. 425–428 (2013). 10.1109/EMBC.2013.6609527 24109714

[28] Martinez, G. V., Serrano, C. A. & Salas, L. ECG baseline drift removal using discrete wavelet transform. In *IEEE International Conference on Electrical Engineering, Computing Science, and Automatic Control*, pp. 1–5 (2011).

[29] Karthikeyan P, Murugappan M, Yaacob S (2012). ECG signal denoising using wavelet thresholding techniques in human stress assessment. Int. J. Electr. Eng. Inform..

[30] Elgendi M, Jonkman M, DeBoer F (2010). Frequency bands effects on QRS detection. PAN.

[31] Almeida LB (1994). The fractional Fourier transform and time-frequency representations. IEEE Trans. Signal Process..

[32] Yaqoob, T., Aziz, S., Ahmed, S., Amin, O., & Alouini, M. S. Fractional Fourier transform based QRS complex detection in ECG signal. In *ICASSP 2020-2020 IEEE International Conference on Acoustics, Speech and Signal Processing (ICASSP)*. IEEE, pp. 931–935 (2020).

[33] Schneider T, Neumaier A (2001). Algorithm 808: Arfit—A matlab package for the estimation of parameters and eigenmodes of multivariate autoregressive models. ACM Trans. Math. Softw. (TOMS).

[34] Zheng, J. *A 12-lead Electrocardiogram Database for Arrhythmia Research covering more than 10,000 Patients* (2019). https://figshare.com/collections/ChapmanECG/4560497/2. 10.1038/s41597-020-0386-x PMC7016169 32051412

[35] Jambukia, S. H., Dabhi, V. K. & Prajapati, H. B. Classification of ECG signals using machine learning techniques: A survey. In *2015 International Conference on Advances in Computer Engineering and Applications*. IEEE, pp. 714–721 (2015).

[36] Evgeniou, T. & Pontil, M. *Support Vector Machines: Theory and Applications* (Springer, 1999).

[37] Zhao, Q. & Zhang, L. ECG feature extraction and classification using wavelet transform and support vector machines. In *2005 International Conference on Neural Networks and Brain*. IEEE, pp. 1089–1092 (2005).

[38] Taravat A, Proud S, Peronaci S, Frate FD, Oppelt N (2015). Multilayer perceptron neural networks model for meteosat second generation seviri daytime cloud masking. Remote Sens..

[39] Sabherwal P, Singh L, Agrawal M (2018). Aiding the detection of QRS complex in ECG signals by detecting S peaks independently. Cardiovasc. Eng. Technol..

[40] Smaoui G, Young A, Abid M (2017). Single scale CWT algorithm for ECG beat detection for a portable monitoring system. J. Med. Biol. Eng..

[41] Pedregosa F, Varoquaux G, Gramfort A, Michel V, Thirion B, Grisel O, Blondel M, Prettenhofer P, Weiss R, Dubourg V (2011). Scikit-learn: Machine learning in Python. J. Mach. Learn. Res..

